# A Poplar Rust Effector Protein Associates with Protein Disulfide Isomerase and Enhances Plant Susceptibility

**DOI:** 10.3390/biology9090294

**Published:** 2020-09-16

**Authors:** Mst Hur Madina, Md Saifur Rahman, Xiaoqiang Huang, Yang Zhang, Huanquan Zheng, Hugo Germain

**Affiliations:** 1 Department of Chemistry, Biochemistry and Physics, Université du Québec à Trois-Rivières, 3351 boulevard des Forges, Trois-Rivières, QC G9A 5H7, Canada; mosammad.hur.madina@uqtr.ca (M.H.M.); md.saifur.rahman@uqtr.ca (M.S.R.); 2 Department of Computational Medicine and Bioinformatics, University of Michigan, 100 Washtenaw Avenue, Ann Arbor, MI 48109, USA; xiaoqiah@umich.edu (X.H.); zhng@umich.edu (Y.Z.); 3 Department of Biology, McGill University, 1205 Dr. Penfield Avenue, Montreal, QC H3A 1B1, Canada; hugo.zheng@mcgill.ca

**Keywords:** fungal rust, effector, GxxxG motif, protein disulfide isomerase, helper protein, plant susceptibility

## Abstract

*Melampsora larici-populina (Mlp)*, the causal agent of *Populus* leaf rust, secretes an array of effectors into the host through the haustorium to gain nutrients and suppress immunity. The precise mechanisms by which these effectors promote virulence remain unclear. To address this question, we developed a transgenic *Arabidopsis* line expressing a candidate effector, Mlp124357. Constitutive expression of the effector increased plant susceptibility to pathogens. A GxxxG motif present in Mlp124357 is required for its subcellular localization at the vacuolar membrane of the plant cell, as replacement of the glycine residues with alanines led to the delocalization of Mlp124357 to the nucleus and cytoplasm. We used immunoprecipitation and mass spectrometry (MS) to identify Mlp124357 interaction partners. Only one of the putative interaction partners knock-out line caused delocalization of the effector, indicating that *Arabidopsis* protein disulfide isomerase-11 (AtPDI-11) is required for the effector localization. This interaction was further confirmed by a complementation test, a yeast-two hybrid assay and a molecular modeling experiment. Moreover, localization results and infection assays suggest that AtPDI-11 act as a helper for Mlp124357. In summary, our findings established that one of *Mlp* effectors resides at the vacuole surface and modulates plant susceptibility.

## 1. Introduction

During infection, plant pathogenic microbes deliver virulence proteins, known as effectors, into host cells to overcome plant immunity and promote parasitic colonization through the manipulation of cellular processes [1]. Once inside host tissues, effectors traffic to various cellular compartments where they interact with host proteins or nucleic acids and exert their virulence function [2,3,4,5,6]. To target these destinations, effectors possess domains or motifs in their sequence; for example, nucleus localized effectors can contain nuclear-localized signals (NLS) and some chloroplast localized effectors may carry a transit peptide [4,7,8]. Uncovering how effector proteins function inside the plant is key to understand pathogenicity mechanisms and to develop more resistant crops [9]. Because investigating pathogenesis on crop species can be challenging, alternative approaches using heterologous expression in *Arabidopsis thaliana* and *Nicotiana benthamina* are extensively used in the functional investigation of effector biology [3,5,10,11,12].

Host proteins that associate with effectors can be categorized as targets or helpers [13]. Target proteins are directly targeted and modulated by effectors to alter host cellular processes. For example, the bacterial effector HopZ1a interacts with positive regulators of immunity to inhibit their activity [14], whereas the *Pseudomonas syringae* T3E AvrB effector binds to the *Arabidopsis* mitogen-activated protein kinase 4 (MPK4), a suppressor of immunity, to induce plant susceptibility [15]. On the other hand, helper proteins may act as co-factors to enable the function, the maturation, or the trafficking of an effector. Less information is available about helpers than about targets so far, perhaps as mutation in helpers may not directly enhance susceptibility to pathogens. For instance, the effectors AvrRPM1 and AvrPto both require myristoylation for their activity, suggesting an interaction with a myristoyl transferase which would serve as a helper for their maturation [16,17]. A well-known helper is the importin-α, a protein whose function is to mediate nuclear entry of NLS-containing proteins; several transcription activator-like (TAL) effectors and Crinklers were shown to require importin-α for their proper nuclear accumulation [7,18,19]. Regardless of whether host proteins act as an effector’s target or helper, they are considered as host susceptibility factors [13].

Rust fungi (Basidiomycetes, Pucciniales) are obligate biotrophic parasites, infect numerous plant families and are the largest group of fungal pathogens [20]. Several rust species are devastating plant pathogens affecting crops and thus food security [21]. *Melampsora larici-populina (Mlp)* causes rust disease on poplar leaves and lead to major yield losses in poplar plantations worldwide [22,23,24]. Although poplar is not a food crop, this pathosystem can be used to better understand other rust pathosystems. Genome and transcriptome analyses have revealed that *M. larici-populina* may have as much as 1184 small secreted proteins (SSPs) [25]. Among these SSPs, candidate secreted effector proteins (CSEPs) have been selected based on features such as expression in poplar leaves during infection and specificity to the Pucciniales order [26,27,28]. When expressed in *N. benthamiana* or *A. thaliana,* several CSEPs of *Mlp* have been shown to accumulate in diverse cell compartments of leaf tissues such as the nucleus, the nucleolus, chloroplasts, mitochondria, and plasmodesmata [5,11]. To date, a number of *Mlp* effectors have been identified as promoting plant susceptibility as determined by *in planta* assays [2,11].

We recently reported that the candidate effector Mlp124357 accumulates in tonoplasts, transvacuolar strands, and bulbs [29]. In this study, we further exploited the *A. thaliana* experimental system to discover that this tonoplast-localized effector affects plant susceptibility to pathogens and we tried to elucidate the mechanisms through which it does so. We used the combined methods of genetics, live-cell imaging, immunoprecipitation, and biochemical analysis to look for the interaction partners of Mlp124357 at the tonoplast. We show that the constitutive expression of this effector increases plant susceptibility to bacterial and oomycete pathogens. We demonstrate that a specific motif of Mlp124357 is necessary for its tonoplast localization and interaction. We also provide evidence through mass spectrometry, a genetic complementation test, a yeast-two hybrid assay (Y2H), and in silico modeling that Mlp124357 associates with a protein disulfide isomerase (PDI) which acts as a helper protein for this effector but not for other *Mlp* effectors having similar numbers of disulfide bridges.

## 2. Materials and Methods

### 2.1. Plants Material and Growth Conditions

*A. thaliana* and *N. benthamiana* plants were grown in soil (AgroMix), in a growth chamber after the seeds underwent a stratification period of 48 h at 4 °C. The plant growth chamber was maintained at 22 °C, 60% relative humidity, and with a 16 h/8 h light/dark cycle. In vitro culture of *Arabidopsis* was performed onto Petri dish containing ½ Murashige and Skoog medium (½ MS) and 0.7% agar. For the selection of the single-insertion homozygous transgenic plants, 15 mg/mL Basta or 50 mg/mL Kanamycin was used.

### 2.2. Cloning Procedures and Plasmid Constructs

The open reading frame (ORF) of Mlp124357 without the portion coding for its signal peptide was obtained from GenScript (Piscataway, NJ, USA). The *PDI-11* coding sequence was amplified from *Arabidopsis.* Amplicons were inserted into the pDONR221 vector (Invitrogen, part of Thermo Fisher Scientific, Waltham, MA, USA) by BP recombination reactions and then into the plant expression vectors pB7FWG2 or pK7WG2 by LR recombination reactions using Gateway technology [30]. All the constructs were sequenced before transformation in *Agrobacterium* C58C1.

### 2.3. Protein Expression in N. benthamiana and A. thaliana

For transient protein expression, constructs were introduced into *A. tumefaciens* strain C58C1 by electroporation and delivered into leaf cells of 4-week-old *N. benthamiana* using the agroinfiltration method previously described [31]. Briefly, recombinant bacterial strains were grown overnight in yeast extract peptone (YEP) medium with spectinomycin (50 mg/L) then harvested, and resuspended into an infiltration buffer (10 mM MgCl_2_ and 150 μM acetosyringone) to obtain 0.5 unit of optical density at 600 nm. One hour after resuspension, leaves were infiltrated on their abaxial side. The agro-infected leaves were collected at 2-days post infiltration for confocal microscopy. Stable *A. thaliana* transgenics were developed by introducing the constructs into *A. thaliana* (Col-0) using the *A. tumefaciens*-mediated floral dip transformation (same strain as used for agroinfiltration) method as previously described [32]. Crossing between the transgenic line expressing the effector and the knockout lines was carried out by following the method as described by Madina et al. [29].

### 2.4. Pathogen Infections Assay

Bacterial infections were performed with 4-week-old *Arabidopsis* plants. *Pseudomonas syringae* pv. *tomato* (*Pst*) DC3000 was grown overnight at 28 °C and infiltrated with a needle-less syringe on the abaxial side of the leaves at 0.001 optical density at 600 nm as previously described by Germain et al. 2018 [11]. *Hyaloperonospora arabidopsidis* (*Hpa*) Noco2 infections were performed with 2-week-old *Arabidopsis* plants using the spray inoculation method described by Dong et al. [33], *eds1-2* allele was used as a control. Statistical significance was determined by Student’s *t*-test (*p* < 0.05) or one-way analysis of variance (ANOVA *p* < 0.05) complemented with Tukey’s HSD test [34].

### 2.5. Membrane Fractionation

The membrane fractionation experiment was carried out according to the method of Widell and Larsson et al. (1981) with the modification applied in Germain et al. (2013) [35,36]. Briefly, a two-phase separation method was used to separate membrane fraction of plant cells, and all preparation steps were maintained at 4 °C or on the ice. 3–5 g of fresh leaves from 3-weeks old Col-0-eGFP and Mlp124357-eGFP were homogenized with a knife blender in the homogenization buffer. The homogenized sample was filtered twice through four layers of cheesecloth and centrifuged at 10,000× *g* for 10 min. Then, the supernatant was transferred to an ultracentrifuge tube and centrifuged at 50,000× *g* for 1 h. The supernatant, containing soluble proteins was discarded and the membrane pellet was resuspended in IP lysate buffer (10 mM MgCl_2_, 50 mM Tris-HCl (pH 7.5), 100 mM NaCl, 0.1% Triton X-100, 1 mM PMSF, and 1X cOmplete protease inhibitor cocktail) [37]. Affinity chromatography of the protein complexes were performed with GFP beads following the protocol by Serino G and Deng XW (2007). Proteins were eluted from the beads by heating the samples at 95 °C for 10 min and analyzed by standard SDS-PAGE followed by Western blotting or mass spectrometry.

### 2.6. Sample Preparation for Mass Spectrometry

Protein digestion and mass spectrometry experiments were performed by the Proteomics Platform of the Centre hospitalier universitaire of the Quebec Research Center, Quebec, Canada. Gel bands of interest were reduced and alkylated then digested with trypsin into peptides prior to mass spectrometry analysis. Peptide samples were injected and separated by LC-MS/MS on a 5600+ triple TOF mass spectrometer (Sciex, Framingham, MA, USA) coupled to an Ekspert NanoLC425 (Sciex). Peptide separation took place on a self-packed picofrit column (New Objective) with reprosil 3u, 120A C18, 15 cm × 0.075 mm internal diameter, (Dr Maisch). Peptides were eluted with a linear gradient from 5–35% solvent B (acetonitrile, 0.1% formic acid) in 35 min, at 300 nL/min. Mass spectra were acquired using a data-dependent acquisition mode using Analyst software version 1.7. Each full scan mass spectrum (400 to 1250 m/z) was followed by collision-induced dissociation of the twenty most intense ions. Dynamic exclusion was set for a period of 12 sec and a tolerance of 100 ppm.

MGF peak list files were created using Protein Pilot version 4.5 software (Sciex). MGF sample files were then analyzed using Mascot (Matrix Science, London, UK; version 2.4.1). Mascot was set up to search against a contaminant database, an *A. thaliana* Uniprot database (82,874 entries) assuming the digestion enzyme trypsin. Mascot analysis was conducted with a parent and fragment ion mass tolerance of 0.10 Da. Carbamidomethyl of cysteine was specified as a fixed modification while deamidation of asparagine and glutamine and oxidation of methionine were specified as variable modifications; 2 missed cleavages were allowed.

Scaffold (version Scaffold_4.8.4, Proteome Software Inc., Portland, OR) was used to validate MS/MS based peptide and protein identifications. Peptide identifications were accepted if they could be established at greater than 5.0% probability to achieve an FDR less than 1.0% by the Scaffold Local FDR algorithm. Protein identifications were accepted if they could be established at greater than 96.0% probability to achieve an FDR less than 1.0% and contained at least 2 identified peptides. Protein probabilities were assigned by the Protein Prophet algorithm [38]. Proteins that contained similar peptides and could not be differentiated based on MS/MS analysis alone were grouped to satisfy the principles of parsimony. All the mass spectrometry results were deposited in the Mass Spectrometry Interactive Virtual Environment (MASSIVE) database and made publicly available using the PXD021290 data identifier.

### 2.7. Western Blot Analysis

The presence of Mlp124357-eGFP, eGFP, and AtPDI-11-eGFP were determined by SDS-PAGE and western blotting. Leaf tissue was harvested from 2–3 week-old stable transgenic plants and protein extracts were prepared as described [39]. The blot was probed with an α-GFP-HRP antibody (1:500 dilution, Molecular Probes, Santa Cruz Biotechnology, Dallas, TX, USA). The bands were revealed with the Clarity^TM^ western ECL substrate (Bio-Rad) according to the manufacturer’s recommendations.

### 2.8. Confocal Microscopy

Small pieces of young leaves from *A. thaliana* or *N. benthamiana* were mounted in water between a slide and a coverslip and were immediately observed. Live-cell imaging was performed on Leica TCS SP8 confocal laser scanning microscope (Leica Microsystems) with a 40X/1.40 oil immersion objective. Images were taken at 1024 × 1024 pixels resolution using line-by-line sequential scanning (when appropriate). The excitation wavelength for eGFP was 488 nm and its emission was collected from 500 to 525 nm. Z-stacks of between 50 and 100 confocal images were acquired and used to generate three-dimensional (3-D) reconstructions using Leica TCS SP8 software (when required). The LAS AF Lite software (Version 3.3) and Adobe Photoshop CS6 were used for the post-acquisition images processing.

### 2.9. RNA Extraction and Transcriptome Analysis

RNA isolation was carried out as described previously [2]. Briefly, total RNA was extracted from 4-day-old *Arabidopsis* plants grown in Petri dishes and quantified before sending for sequencing. Ion Torrent Technology was used for library construction and sequencing (Université Laval, Quebec City, Canada). Transcriptomics data were processed as reported previously [2] and all transcriptomic data were submitted to Genbank under project ID PRJNA608508. Gene Ontology (GO) enrichment of both up- and down-regulated genes (having Q-value ≤ 0.05 and a fold-change ≥ 3) were investigated using the Cytoscape software (version 3.1.1) with the plug-in ClueGO and CluePedia [40].

### 2.10. Y2H Reporter Assays

Coding sequences of Mlp124357 and *AtPDI-11* without their signal peptide were cloned into pGBKT7 (binding domain) and pGADT7 (activation domain), respectively, by homologous recombination in yeast strain Y187 or Y2H gold. Bait protein-encoding vector pGBKT7 expressing Mlp124357 and the prey protein-encoding vector pGADT7 expressing AtPDI-11 were transformed into the yeast strain Y2H gold according to the Clontech Y2H protocol. Transformants were plated along with a negative control onto yeast synthetic double dropout medium (DDO) lacking Leu and Trp and quadruple dropout selective medium (QDO) lacking Trp, Leu, His, and Ade (Takara, Mountain View, CA, USA) and incubated at 30 °C for 3 to 4 days. For photographing, series of dilution (10^−0^, 10^−1^, 10^−2^) were prepared for each transformant and 10 µL were placed onto DDO and QDO medium and incubated at 30 °C for 3 to 4 days.

### 2.11. Molecular Modeling of the Proteins

Three-dimensional structures of the Mlp124357 were produced through homology modeling using the online tool QUARK [41]. AtPDI-11 and PtPDI structure homology-modeling was obtained from sequence alignment against the homology template of Protein disulfide-isomerase A3 (PDB: 6eny) using I-TASSER [42]. The binding efficiency of the effector to AtPDI-11 or PtPDI was determined using four different protein-protein docking servers Cluspro, Grammx, Patchdock, and ZDock [43,44,45,46]. The generated protein-protein complexes were visualized and analyzed through PyMOL [47,48].

## 3. Results

### 3.1. Selection and Phylogenetic Analysis of Mlp124357

**Figure 1:**
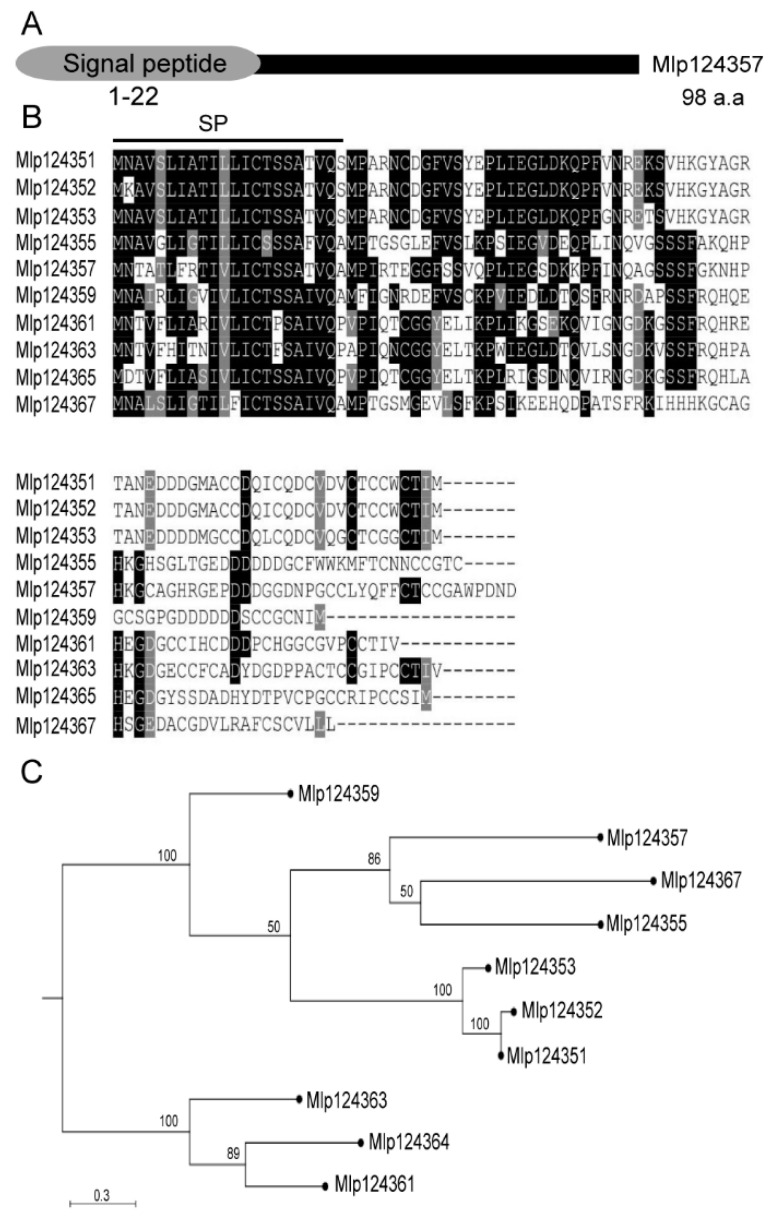
Selection and phylogenetic analysis of Mlp124357. (**A**) Schematic representation of protein topology of Mlp124357. N-terminus of Mlp124357 contains a secretory signal peptide (SP) (**B**) Multiple sequence alignment of the ten effector proteins that are the members of the *M. larici-populina* CPG4890 SSP family. Predicted signal peptides (SP) are marked with a line. Black boxes indicate conserved residues and grey boxes indicate similar residues. (**C**) Phylogenetic tree of the CPG4890 gene family, obtained with CLC workbench using the Kimura protein distance value and neighbor-joining tree method. Bootstrap values are indicated.

The analysis of *M. larici-populina’s* genome revealed more than one thousand potential small-secreted proteins [25]. To select candidate secreted effector proteins for functional investigation, we followed various criteria that were previously described [5,11]: these included that the sequences must be of small size, possess a signal peptide and conserved cysteines, and must not have conserved sequences outside the order Pucciniales; they must also be detected in infection structures. One of the small secreted peptides that met these criteria is Mlp124357. It belongs to the family CPG4890, which contains 10 members and appears to be under positive selective pressure [27]. Each member carries an N-terminal signal peptide region (amino acids 1–22 in the case of Mlp124357) and encodes a short peptide 80–98 amino acids long (molecular weights 9–10 kDa) (Figure 1A,B).

The sequence identity ranges from 40–60% between Mlp124357 and the other family members (Figure 1C). In addition, this peptide contains eight cysteine residues, and its expressed sequence tag (EST) has not been detected in spores but is abundant in infected poplar leaves, supporting infection specific expression. With the exception of the members of its close family, Mlp124357 shows little similarity to other proteins, whether in Pucciniales or any other genus, indicating that it is a fairly unique protein in all kingdoms. Hence, the infection specific expression of Mlp124357 and its uniqueness triggered us to investigate its localization and role *in planta* during infection.

### 3.2. Mlp124357 Expression in Planta Affects the Plant Susceptibility to Bacterial and Oomycete Pathogens

**Figure 2:**
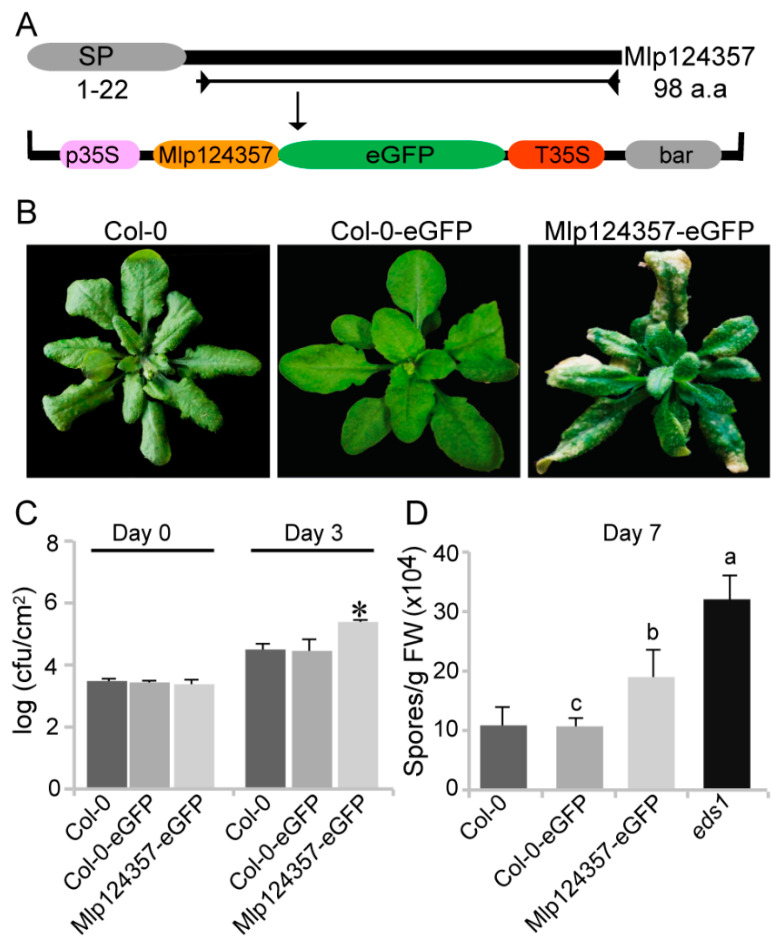
Mlp124357 expression *in planta* affects the plant susceptibility to bacterial and oomycete pathogens. (**A**) Schematic representation of the T-DNA construct used for *in planta* expression of the Mlp124357 mature coding sequence. (**B**) Morphology of wild-type (Col-0) expressing eGFP or Mlp124357-eGFP. Photographs were taken from 4-week-old soil-grown plants. (**C**) Growth of PstDC3000 bacteria in *Arabidopsis*. Leaves of each genotype were infiltrated with a *Pst*DC3000 bacterial suspension (OD600 = 0.001) and the bacterial growth was measured on day 0 and day 3 after infection. Statistical significance was evaluated using a Student’s *t-test* (*p* < 0.05); asterisk indicates a statistically significant difference between plants carrying effector and Col-0-GFP. Five replicates were used for each genotype; cfu, colony-forming unit. (**D**) Growth of *H. arabidopsidis* Noco2 in *Arabidopsis*. Each genotype was spray inoculated with *H. arabidopsidis* Noco2 spores (20,000 conidiospores/mL) and the number of conidiospores was quantified 7 days after inoculation. Statistical significance was evaluated using ANOVA (*p* < 0.05) with Tukey’s test. Letters denote a significant difference between Col-0-eGFP, Mlp124357eGFP, and *eds1*. FW, fresh weight. Both bacterial and oomycete infection experiments were repeated at least three times and representative data are shown.

To express Mlp124357 and determine its localization *in planta*, we cloned its coding sequence without the signal peptide tagged with enhanced Green Fluorescent Protein (eGFP) under the control of a CaMV35S promoter in a Gateway expression vector (Figure 2A). Then, we expressed the Mlp124357 fusion construct in wild-type *Arabidopsis* (Col-0) as a stable transgenic line and also developed a control line expressing eGFP under the control of the same promoter. Figure S1 shows that both proteins (eGFP and Mlp124357-eGFP) were expressed at the appropriate molecular weight. This result confirms that Mlp124357-eGFP is expressed *in planta*, is not further processed, and thus can be used for further investigations. The resulting transgenic plants are shown in Figure 2B. The plants expressing only eGFP are indistinguishable from the wild-type whereas the plants overexpressing Mlp124357-eGFP display narrower leaves, darker green leaves, chlorosis, and drying of the leave tips (Figure 2B).

To evaluate if Mlp124357 could interfere with the plant susceptibility to pathogens, we subjected control plants and plants expressing Mlp124357 to bacterial and oomycete pathogens. The plants expressing Mlp124357 harbored nearly 10-fold more *Pseudomonas syringae* pv. *tomato* DC3000 bacteria after three days than control plants, indicating that they are hypersusceptible to this bacterial hemibiotrophic pathogen (Figure 2C). As rusts cannot infect *A. thaliana*, we used *H. arabidopsidis* for our infection assay. Although *H. arabidopsidis* is not a rust (it is an oomycete), it is also an obligate biotrophic pathogen that infects plants by making haustoria. Seven days after inoculation, we quantified the number of spores and detected a significant increase in the susceptibility of Mlp124357-eGFP transgenic plants compared to Col-0 (*p* < 0.05) (Figure 2D), but not as pronounced as the one observed in the hypersusceptible mutant line *eds1,* indicating that the effector does not make the plant as susceptible as the *eds1-2* mutation.

### 3.3. Mlp124357 Possesses a GxxxG Motif that Is Required for the Interaction with Tonoplasts

**Figure 3:**
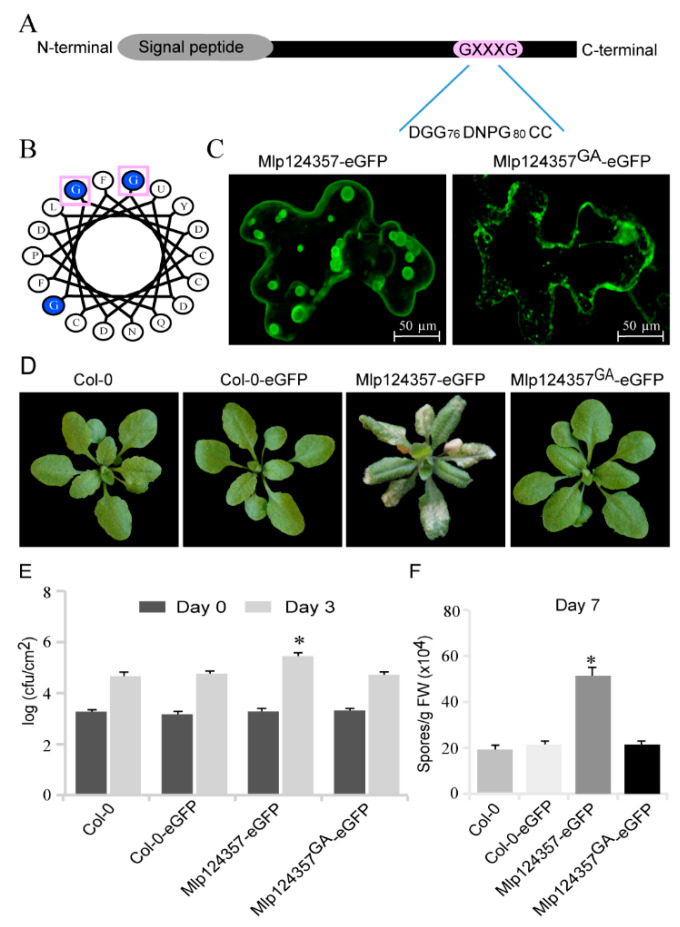
Mlp124357 possesses a GxxxG motif that is required for the interaction with tonoplasts. (**A**) Schematic representation of the GxxxG motif in the C-terminus region of Mlp124357. (**B**) A predicted helical wheel projection of Mlp124357. The glycine residues at positions 76 and 80 are indicated by square boxes. (**C**) Fluorescence imaging of *N. benthamiana* cells expressing eGFP fusions of Mlp124357 and Mlp124357 ^GA^ mutant at 2 dpi using confocal microscopy of epidermal cells. (**D**) Morphology of each genotype. Photographs are from 4-week-old soil-grown plants. (**E**) Leaves of each genotype were infiltrated with a PstDC3000 bacterial suspension at OD 600 = 0.001 and bacterial growth was quantified in colony-forming units (cfu) on day 0 and day 3 after infection. One-way ANOVA (*p* < 0.05) with Tukey’s test was performed. The asterisk indicates a statistically significant difference between Col-0-eGFP, Mlp124357-eGFP, and Mlp124357GA-eGFP. Five replicates were used for each genotype. (**F**) Each genotype was spray inoculated with *H. arabidopsidis* Noco2 spores (20,000 conidiospores/mL) and the number of conidiospores was quantified 7 days after inoculation. Statistical significance was evaluated using one-way ANOVA with Tukey’s test (significance set at *p* < 0.05). The asterisk denotes a significant difference between Col-0-eGFP, Mlp124357-eGFP, and Mlp124357GA-eGFP. FW, fresh weight. Both bacterial and oomycete infection experiments were repeated at least three times and representative data are shown.

We have previously reported that Mlp124357 targets the tonoplasts *in planta* [29]. However, Mlp124357 does not possess any transmembrane domain. Thus, we rationalized that Mlp124357 may interact with an integral tonoplast protein. We investigated the primary sequence of Mlp124357 and discovered a GxxxG motif (Figure 3A), which is known for mediating interactions with membrane proteins [49,50]. The secondary structure of the Mlp124357 sequence was modeled as an alpha-helical wheel projection using helix prediction software (http://kael.net/helical.htm). The two glycines of the GxxxG motif at positions 76 and 80 were found on the same side of the α-helix (Figure 3B, pink box). This amino acid orientation of the GxxxG motif can be involved in protein interactions between membrane proteins [51].

To investigate whether the two glycines are implicated in Mlp124357 localization, we carried out site-directed mutagenesis to substitute them into alanines (Ala). We generated a mutant construct Mlp124357^G76A-G80A^ fused in C-terminal to eGFP, which is hereafter referred to as Mlp124357^GA^. The fusion protein was transiently expressed in *N. benthamiana* leaves and its subcellular localization was assessed by confocal laser microscopy. The expression of wild type Mlp124357-eGFP was still detected in tonoplasts and bulbs of epithelial cells (Figure 3C). However, the replacement of the glycine residues with alanines led to the delocalization of the effector (Figure 3C).

Subsequently, to test whether the delocalization of Mlp124357 affects the activity of the effector, we developed a stable *Arabidopsis* transgenic line (Figure 3D) with the glycine mutant construct. The glycine mutant line no longer displayed the chlorotic phenotype on the leaves; rather, it was indistinguishable from WT plants (Figure 3D). We then confirmed that Mlp124357^GA^ localization in *A. thaliana* correlated with what was observed in *N. benthamiana,* which is that Mlp124357-eGFP accumulates in the tonoplast, transvacuolar strands, and bulbs while Mlp124357^GA^ accumulates in the nucleus and cytoplasm (Figure S2). Further, these transgenic lines were used to perform infection assays to evaluate their susceptibility to pathogens. The plants expressing Mlp124357^GA^ showed a level of bacterial growth that was similar to control plants Col-0 and Col-0-eGFP (Figure 3E). Similarly, inoculation experiment with *H. arabidopsidis* spores demonstrated wild-type like susceptibility of Mlp124357^GA^-eGFP compared to Mlp124357-eGFP (*p* < 0.0001) (Figure 3F). From these experiments, we conclude that the GxxxG motif is required for the localization and the function of the Mlp124357 effector.

### 3.4. Protein Disulfide Isomerase 11 as a Potential Plant Interactor of Mlp124357

**Figure 4:**
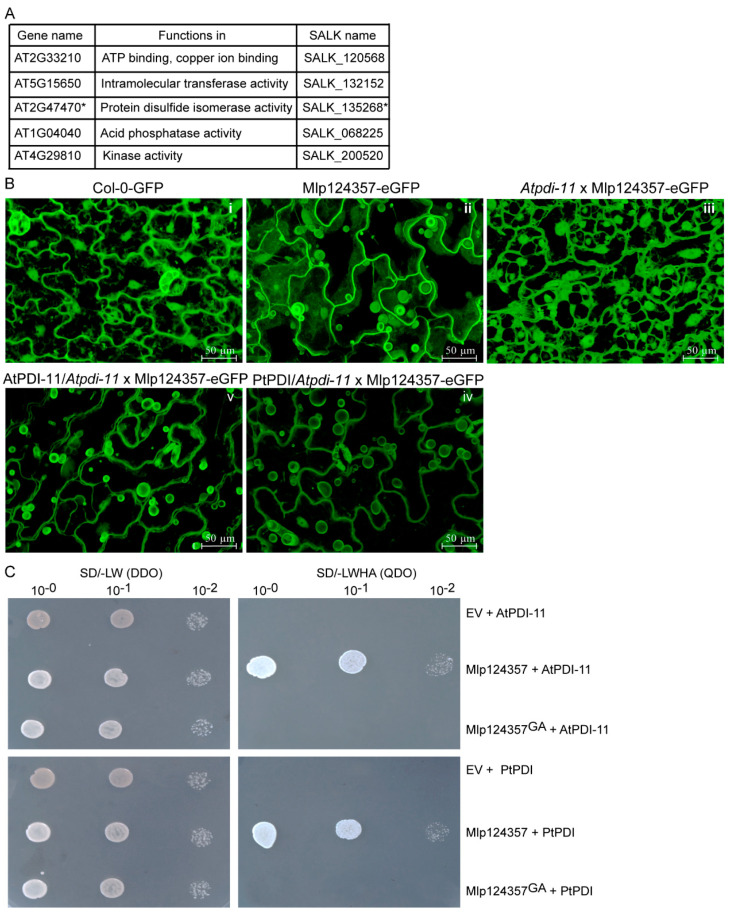
Protein Disulfide Isomerase 11 as a potential plant interactor of Mlp124357. (**A**) The five tonoplast-localized proteins selected from the IP/MS list. (**B**) Interaction between Mlp124357 and AtPDI-11 or PtPDI was detected by a genetic analysis/complementation test. Live-cell imaging of leaf epidermal cells of each genotype. (**C**) Interaction between Mlp124357 and AtPDI-11 or PtPDI was evaluated by Y2H. The plates were photographed 2 days after inoculation.

Since the Mlp124357 peptidic sequence lacks a transmembrane domain and that no post-translational modification (myristoylation, acetylation, or mannosylation) could be predicted, we rationalized that the small secreted peptide may interact with a plant protein to achieve its tonoplastic localization. To identify putative interaction partners of Mlp124357 in membranes, we first assessed if it could be isolated from membrane enriched fractions. It appears that Mlp124357’s association with the membranes is not very strong as it could also be detected in the supernatant of the membrane fraction (Figure S3). However, since Mlp124357 was mostly retained in membranes, we immunoprecipitated it from the membrane fraction using anti-GFP beads and subjected SDS-PAGE gel bands to mass spectrometry to identify potential plant protein interactors. From the MS analysis, 40 *A. thaliana* proteins were identified as potential interactors of the Mlp124357 effector (Table S1). However, since Mlp124357 accumulates in the tonoplasts, we only selected five proteins predicted to be tonoplastic for further investigation (Figure 4A).

To verify the potential interacting protein partners, we crossed knockout (KO) lines of four of those five tonoplasts proteins (line SALK-200520 could not be made homozygous) with the Mlp124357-eGFP transgenic line and then assessed Mlp124357-eGFP localization using confocal microscopy (Figure 4A). We hypothesized that if one of those proteins was an interactor serving as an anchor protein to Mlp124357 at the tonoplasts, the effector would change localization in the knockout line. Interestingly, the absence of one tonoplast protein named protein disulfide isomerase 11 (*PDI-11*; gene ID: *AT2G47470*) led to the delocalization of Mlp124357-eGFP (Figure 4B and Figure S4). We confirmed that the delocalization was caused by the absence of PDI-11 by complementing the knockout line with the wild-type *Arabidopsis PDI-11* sequence, which restored Mlp124357-eGFP’s localization. As *Mlp*’s host is poplar, we also assessed by genetic complementation if the effector could interact with *Populus trichocarpa* PDI (AtPDI-11and PtPDI share about 87% sequence identity (Figure S5). We observed that both *Arabidopsis PDI-11* and poplar *PDI* could restore the tonoplastic localization of Mlp124357-eGFP in the *pdi-11* KO background (Figure 4B panels IV and V). In addition, to verify the cellular localization of AtPDI-11 or PtPDI, both were cloned in fusion with eGFP (AtPDI-11-eGFP or PtPDI-eGFP), transiently overexpressed in *N. benthamiana* leaf cells by *Agro*-infiltration and observed by confocal microscopy. The eGFP signal suggested a localization in both the tonoplasts and ER structures (Figure S6). Overall, these results suggest that PDI is required for the localization of Mlp124357 at the tonoplasts. We also assessed whether AtPDI-11 or PtPDI were in frame with the eGFP using immunoblot and found that both proteins are at the expected molecular weight (Figure S1).

Further confirmation of the interaction between Mlp124357 and AtPDI-11 or PtPDI was carried out using a Y2H assay. All construct combinations could grow on the double dropout media, indicating that the yeast had received both plasmids in all cases (Figure 4C left panel). Independent co-transformation experiments showed that yeast co-expressing a bait-Mlp124357 construct with a prey-AtPDI-11 or a prey PtPDI construct was able to grow on quadruple dropout medium (Figure 4C), whereas the bait-Mlp124357^GA^ construct did not grow (Figure 4C) when coexpressed with *Arabidopsis* or poplar PDI. The quadruple dropout media will enable the growth of the yeast only if there is a protein-protein interaction between the activation and binding domain of the GAL4 transcription factor. The immunoprecipitation, the complementation, and the Y2H results provide evidence that Mlp124357 associates with protein disulfide isomerase 11, which is required for the localization of the effector *in planta*.

### 3.5. Molecular Modeling also Supports the Association of AtPDI-11 and Mlp124357 Effector

**Figure 5:**
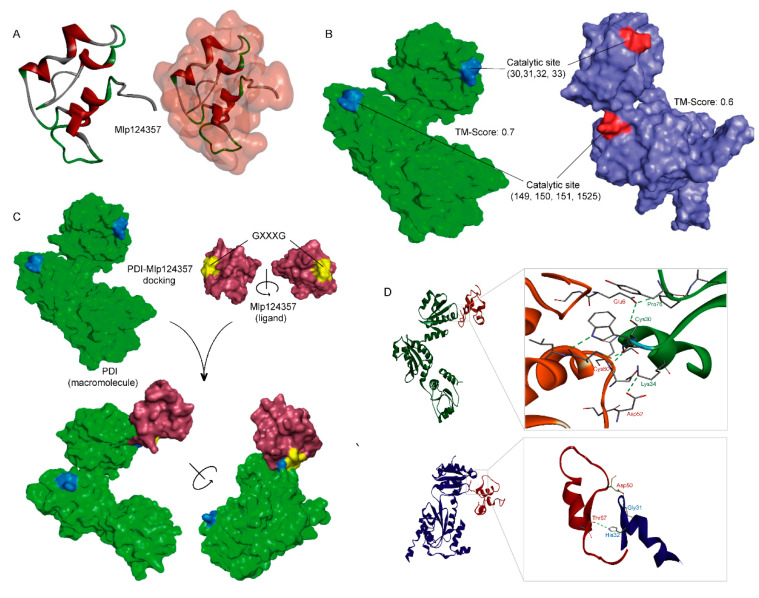
Molecular modeling also supports the association of AtPDI-11 and Mlp124357 effector. (**A**) Ab initio structure of Mlp124357. (**B**) Predicted structure and catalytic sites of PtPDI (left-green) and AtPDI-11 (right-blue). TM-scores have values between 0 and 1, where 1 indicates a perfect match between two structures. (**C**) Functional approach of docking between Mlp124357 and PtPDI. (**D**) The orientation and interactions of PtPDI-Mlp124357 (upper panel) and AtPDI-Mlp124357 (lower panel) complexes.

To further assess the interaction between PDI and the effector, we sought to use protein structures modeling. The three-dimensional (3D) structure of Mlp124357 was built through the ab initio protein structure prediction software QUARK [41] (Figure 5A) because, as we mentioned earlier, Mlp124357 is a unique effector and no good template can be identified through template-based structure modeling, i.e., I-TASSER [52]. However, the 3D structure models of both AtPDI-11 and PtPDI were constructed using I-TASSER, with estimated template modeling scores (TM-score) of 0.6 and 0.7, respectively (Figure 5B).

Protein-protein docking was performed between Mlp124357 and AtPDI-11 or Pt-PDI using different methods (e.g., Z-DOCK, ClusPro, PatchDock [43,44,45]). The top-ranked docking results from different servers showed similar binding poses of Mlp124357 bound to AtPDI-11 or PtPDI (Figure 5C; a representative pose from Z-DOCK). Common catalytic residues (CYS_30/149,_ GLY_31/150,_ HIS_32/151,_ and CYS_33/152_) are found in both AtPDI-11 and PtPDI (Figure 5B) and they are involved in well-established hydrogen bonding networks with Mlp124357 (Figure 5D). Hence the molecular modeling seems to support the robust experimental data obtained so far and gives some insights on how the effector protein Mlp124357 interacts with its binding partner (e.g., AtPDI-11 and PtPDI).

In addition, to assess if the previously observed delocalization and phenotype suppression observed with the Mlp124357^GA^ mutant is caused by impaired protein folding, generating a misfolded non-functional protein, we subjected the Mlp124357^GA^ mutant to the same modeling (Figure S7). The results indicate that the change of GXXXG to AXXXA causes a similar albeit different folding of Mlp124357 (RMSD: 7.23, TM: 0.3) which is sufficient to disrupt the binding affinity to the PDI catalytic site.

### 3.6. The Mlp124357-PDI Association Takes Place in an Effector-Specific Manner

**Figure 6:**
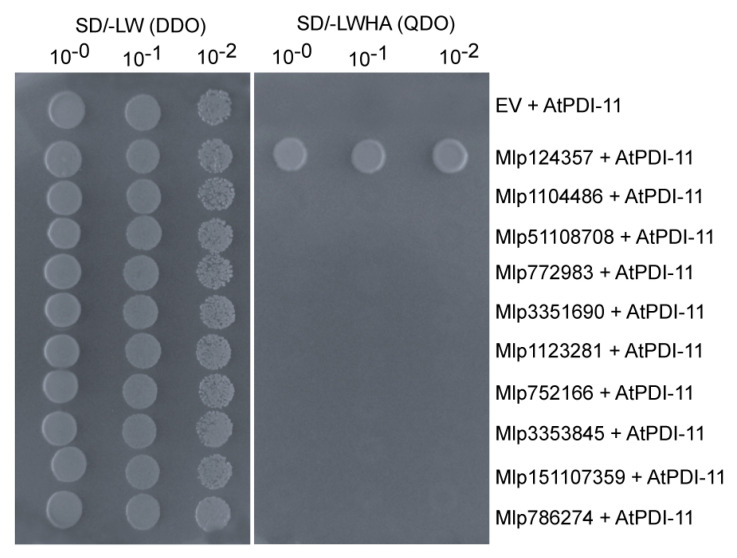
The Mlp124357-PDI association takes place in an effector-specific manner. Co-expression of a prey construct containing AtPDI-11, with Mlp124357, Mlp1104486, Mlp51108708, Mlp772983, Mlp3351690, Mlp1123281, Mlp752166, Mlp3353845, Mlp151107359, or Mlp786274 as baits in yeast to test interaction between PDI and effectors containing multiple cysteines. Yeast co-expressing the indicated combination of bait and prey were spotted on the synthetic double dropout medium lacking leucine and tryptophan (SD/-LW (DDO)) to show the expression of both constructs and quadruple dropout medium lacking leucine, tryptophan, histidine, and adenine (SD/-LWHA (QDO)) to reveal an interaction. Only yeast co-expressing AtPDI-11 and Mlp124357 grew on SD/-LWHA plates. The plates were photographed 2 days after inoculation.

As protein disulfide isomerases catalyze the formation of a disulfide bond between cysteine residues within proteins and as most effector proteins contain several cysteine residues (normally 5–8), we considered that the interaction with PDI-11 could be a general interaction between all (or many) effectors and PDI-11. To determine whether the interaction between Mlp124357 and AtPDI-11 was specific or general, we use the yeast two-hybrid system to assess the interaction between AtPDI-11 prey and eight *Mlp*-effectors also having several predicted disulfide intramolecular bridges (Mlp1104486, Mlp51108708, Mlp772983, Mlp3351690, Mlp1123281, Mlp752166, Mlp3353845 and Mlp151107359). The yeasts co-expressing the effectors with AtPDI-11 did not grow on quadruple dropout medium, like the negative control (Figure 6). This result suggests that the interaction between PDI-11 and Mlp124357 is specific and that PDI-11 does not serve as a general effector maturation interactor.

### 3.7. Protein Disulfide-Isomerase 11 Acts as a Helper of Mlp124357

**Figure 7:**
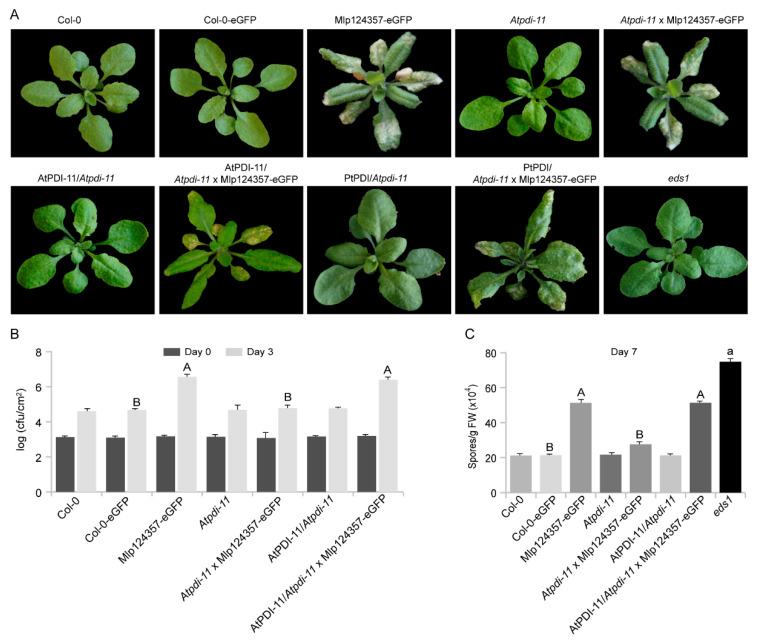
PDI-11 is not involved in plant immunity. (**A**) Morphology of each genotype that were used for the infection assays. Photographs were taken from 4-week-old soil-grown plants. (**B**) Growth of PstDC3000 bacteria in *Arabidopsis*. One-way ANOVA (*p* < 0.05) with Tukey’s test was performed to compare genotypes with GFP or without GFP. There was no difference between genotype without GFP. Different letters indicate statistically significant difference between Col-0-eGFP, Mlp124357-eGFP, Atpdi-11 x Mlp124357-eGFP, and AtPDI-11/Atpdi-11 x Mlp124357-eGFP. For each genotype five replicates were used; cfu, colony-forming unit. (**C**) Growth of *H. arabidopsidis* Noco2 oomycete in *Arabidopsis*. For genotypes without eGFP (Col-0, Atpdi-11, AtPDI-11/Atpdi-11, and *eds1*), significance was evaluated using ANOVA (*p* < 0.05) with Tukey’s. For genotypes with eGFP (Col-0-eGFP, Mlp124357-eGFP, Atpdi-11 × Mlp124357-eGFP and AtPDI-11/Atpdi-11 × Mlp124357-eGFP) significance was evaluated using ANOVA (*p* < 0.05, different capital letters denote significant difference) with Tukey’s test. FW, fresh weight. Both bacterial and oomycete infection experiments were repeated at least three times and representative data are shown.

To assess whether AtPDI-11 is a target or a helper of Mlp124357, we conducted infection assays with two different pathogens (*Pst*DC3000 and *H. arabidopsidis*) on plants expressing PDI-11 or not. It should be noted that the leaf phenotypes of *Atpdi-11* and AtPDI-11/*Atpdi-11* were similar to control plants Col-0 or Col-0-eGFP, but in the presence of the Mlp124357 effector, the *Atpdi-11,* AtPDI-11/*Atpdi-11,* and PtPDI/*Atpdi-11* lines exhibited chlorotic symptoms on their leaves (Figure 7A). This result suggests that, although the effector is mislocalized in the *pdi-11* line, it seems to retain its phenotype-altering activity.

Compared to the control wild-type Col-0 or Col-0-eGFP, the *Atpdi-11* and AtPDI-11/*Atpdi-11* lines showed similar pathogen growth (Figure 7B,C). This result suggests that PDI-11 does not contribute significantly to the plant immunity and that Mlp124357 is unlikely to target it to increase the pathogen virulence. As shown previously, Mlp124357 promoted bacterial and oomycete growth, but interestingly it was unable to do so in the absence of AtPDI-11. As such, AtPDI-11 appears to be required for the localization and the virulence activity of Mlp124357, although the effector’s capacity to affect the global plant phenotype appears to be uncoupled from its virulence activity. Moreover, both *P. syringae* and *H. arabidopsidis* are pathogens that translocate effectors into *Arabidopsis*. The fact that their virulence in a *pdi-11* KO line is similar to the control (Col-0 or Col-0-eGFP) further supports that AtPDI-11 is not required for the maturation of other effectors than Mlp124357, as if *H. arabidopsidis* or *P. syringae* effectors would require AtPDI-11 for their maturation, a decrease in pathogen growth would be observed in the KO line, which is not the case.

### 3.8. Transcriptome Analysis of the Arabidopsis Transgenics Line Expressing Mlp124357

**Figure 8:**
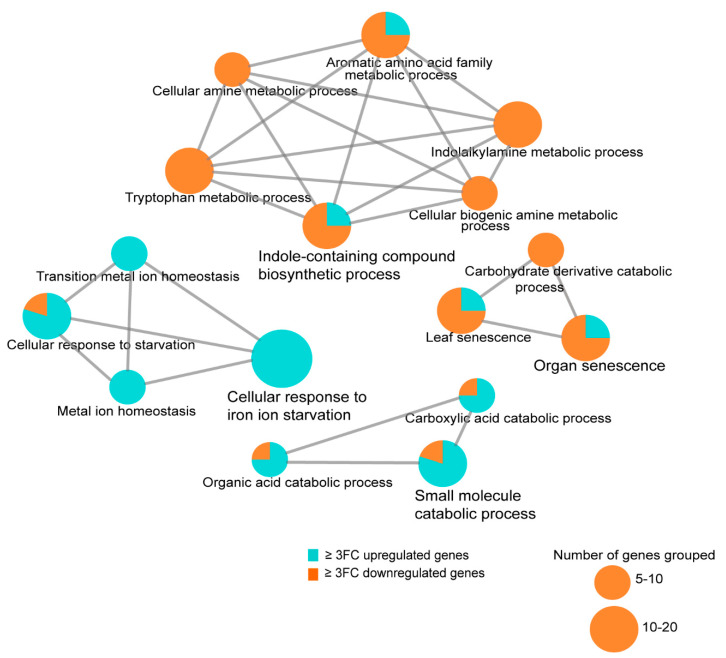
Transcriptome analysis of the *Arabidopsis* transgenic line expressing Mlp124357. Expression of Mlp124357 in *Arabidopsis* deregulates groups of genes associated with senescence, Fe homeostasis, and fungus defense. GO term enrichment analysis was performed with both up and deregulated genes filtered by Q-value ≤ 0.05 and fold-change ≥ 3 using the Cytoscape software (version 3.1.1). ClueGO plug-in of Cytoscape was used to visualize enriched functions for both up- and down-regulated genes.

To identify host molecular pathways altered by Mlp124357, we performed a genome-wide gene expression analysis using RNA-seq of 4-day-old *A. thaliana* Mlp124357 stable transgenic line and control plants expressing eGFP. In the Mlp124357 transgenic line, 268 and 353 genes were up- and down-regulated by 2-fold change or greater, respectively, in comparison with control plants (Table S2). Next, we performed GO term enrichment analysis of genes that were 3-fold change up- and down-regulated in plants expressing Mlp124357 to determine the relevant biological processes altered by Mlp124357. Four functional groups of significantly enriched GO terms were noted: cellular response to iron ion starvation, indole-containing compound biosynthetic process, organ senescence, and small molecule catabolic process (Figure 8).

Remarkably, multiple defense-related genes such as YLS9, CHI, CYP79B2, BGLU21 were down-regulated in the Mlp124357-eGFP transgenic line compared to the eGFP transgenic plants. Several genes coding for BHLH transcription factors, for example, BHLH038, BHLH039, and BHLH040, which are involved in the plant Fe deficiency response, as well as GLK2, which is involved in plant susceptibility to fungus, were up-regulated in the Mlp124357-eGFP transgenic line (Figure 8). These results support our observations of leaf chlorosis (Figure 2C) and plant susceptibility to pathogens (Figure 3D,E).

## 4. Discussion

Effectors play a key role in plant-microbe interactions [53] and many of them are shown to act as virulence factors that are capable to suppress plant defense responses and enhance pathogenesis [54]. However, little is known about the effector functions of *Melampsora larici-populina,* a devastating pathogen of poplars worldwide. In this study, we used *A. thaliana* and *N. benthamiana* as heterologous systems and a poplar leaf rust effector, Mlp124357, as a probe to understand the potential function of this *Mlp* effector in plants. We identified PDI-11 as a putative helper of Mlp124357, defined their specific physical interaction both in yeast and *in planta,* and showed that this interaction between Mlp124357 and PDI-11 is required for the increased plant susceptibility to pathogens. Specifically, we identified a GxxxG motif in Mlp124357 that mediates its interaction with PDI-11. Normally, Mlp124357 accumulates in the tonoplasts, bulbs, and transvacuolar strands; however, when the two glycines of the GxxxG motif were replaced by alanines, the Mlp124357^GA^ mutant lost it’s *in planta* tonoplastic localization and was no longer able to enhance pathogen growth. To our knowledge, a connection between rust pathogens and the tonoplasts has not previously been established.

When *Arabidopsis* was exposed to pathogens with different lifestyles (e.g., bacteria and oomycetes), we observed that Mlp124357 enhanced pathogen growth. Both *Hpa* and *Mlp* are obligate biotrophic filamentous pathogens of dicot plants and possess a similar infection strategy in leaf tissues. However, Mlp124357 also increased the growth of a bacterial pathogen (*Pst*). Therefore, we conclude that Mlp124357 alters a host mechanism that is active against both bacteria and biotrophic filamentous pathogens.

The morphology of plants expressing Mlp124357 is altered, e.g., the plants show narrower leaves, chlorosis, and yellowing of the leaf tips (Figure 2 and Figure 7). Expression of bacterial as well as filamentous pathogen effectors can affect the host immune response and induce a variety of phenotypes in plants, including chlorosis [55,56]. For instance, fungal effectors SnTox [57,58] and FvTox1 [59] as well as bacterial effectors AvrB [60] and HOPQ-1 [61] induce chlorosis symptoms in plants. A chlorotic phenotype has previously been reported to correlate with plant susceptibility [62], thus induction of both chlorosis and plant susceptibility by Mlp124357 can reflect a genuine effector activity. However, when we mutated the glycine residues of the GxxxG motif of Mlp124357 and expressed it in *Arabidopsis*, we could no longer observe leaf chlorosis, suggesting that adequate protein localization is required for the chlorosis-inducing activity of the effector. In addition, the in silico folding of the effector suggests that the AxxxA mutant adopts a very similar folding to the wild-type effector, suggesting that, although it no longer interacts with PDI-11, the protein would still be stable. On the other hand, Mlp124357 was still capable of inducing chlorosis in *pdi-11* KO plants, but was unable to induce plant susceptibility in these plants, suggesting that virulence and chlorosis can be uncoupled. One possible explanation is that the interaction with PDI-11 is required for a subsequent interaction with an unidentified virulence target involved in conferring susceptibility to pathogens, but that the effector also has a second target which can be reached independently of the interaction with PDI-11.

Recently, we reported that Mlp124357 localizes to tonoplasts, bulbs, and transvacuolar strands [29]. However, how the *Mlp* pathogen benefits from manipulating the host tonoplasts and vacuolar substructures is unclear [63]. An effector of *Hpa,* HaRxL17, was also reported to localize to tonoplasts and to enhance plant susceptibility [3]. Therefore, pathogen effectors may target the tonoplasts to modulate/suppress vacuole-mediated defense responses. Further mechanistic investigations of the pathogen effector/tonoplasts interplay should shed light on the biological significance of this phenomenon.

We identified Protein Disulfide Isomerase-11, a member of the Protein Disulfide Isomerase (PDI) gene family, as a plant interactor of Mlp124357 in *Arabidopsis*. The major function of PDIs in plants is to facilitate the formation of disulfide bonds by induction, oxidization, and isomerization; all of which are essential for the proper folding and maturation of proteins. For example, HSP70, HSP90, and DNAJ-like proteins have been shown to perform similar functions as chaperones in plant-pathogen interaction [64]. Like other effectors, cysteine-rich Mlp124357 is expected to translocate as an unfolded protein to the plant cell, thus, we speculated that a PDI could be recruited by *Mlp* to act as a cellular chaperone during infection. The identification of AtPDI-11 or PtPDI as mediators of *Mlp* virulence activity suggests that other members of the PDI gene family might play a similar role in other plant-rust interactions; however, our evidence suggests that PDI-11 is not a generic interactor for all *Mlp* effectors since it did not interact with any of the other effectors tested. Additionally, the *pdi-11* line did not exhibit a pathogen resistance phenotype, which would have been the case if the assayed pathogens’ effectors would not have been able to mature as a result of the absence of a universal maturation PDI protein.

Variation has been reported in the subcellular localization of PDIs in *Arabidopsis* [65]. *A. thaliana* encodes 12 PDIs, which are classified in three subfamilies, PDI-11 is the only member of the PDI-D subfamily and it is also the only *Arabidopsis* PDI lacking an ER retention signal [65]. Most PDIs were shown to localize to the ER lumen [66,67,68], while several PDI isoforms have been localized to other cellular organelles such as Golgi, chloroplasts, nucleus, and tonoplasts [66,67,68]. Moreover, functional human PDIs are also demonstrated to accumulate at the cell surface, the extracellular space, the cytosol, and the nucleus [69]. Our result indicates that AtPDI-11 accumulates both at the ER and tonoplasts and interacts with Mlp124357. However, in the *pdi-11* knockout line which expressed Mlp124357, we observed that Mlp124357 was delocalized to the cytosol and nucleus. This result strongly suggests that the interaction between the two proteins occurs on the cytosolic side of the tonoplasts rather than on the luminal side of the tonoplasts.

Some host proteins that are targeted by pathogen effectors can be “helpers” who enable effector functions, while others are “targets” [70]. Previous studies demonstrated that the folding of a pathogen’s effector facilitated by host protein might be important to modulate this effector’s functions in host cells during pathogenesis. For instance, cyclophilin ROC1 (*Arabidopsis*) and GmCYP1 (soybean) are required to activate the bacterial effector AvrRpt2 [71,72] and the fungal effector Avr3b [73], respectively. Our study demonstrated that PDI absence by itself does not affect plant susceptibility, suggesting that PDI is not directly involved in immunity but may be responsible for the activation of the *Melampsora larici-populina* effector Mlp124357. In other words, AtPDI-11 is likely the host helper recruited by Mlp124357 to enhance plant susceptibility. These results suggest that recruitments of host factors as “helper” is a common pathogenesis mechanism shared by pathogens.

The molecular interaction of poplar with the economically important pathogen *Melampsora larici-populina* remains largely unknown. This work can essentially increase our knowledge of the poplar-*Melampsora larici-populina* pathosystem as well as highlight the importance of helper proteins as susceptibility factors. Future work will be directed toward understanding whether Mlp124357 has other target proteins in plant cells and uncovering the specific mechanism by which Mlp124357 affects plant defense, which could help plan control strategies of rust pathogens.

## Supplementary Materials

The following are available online at https://www.mdpi.com/2079-7737/9/9/294/s1, Figure S1: Immunodetection of eGFP protein in wild-type (Col-0) and stable transgenic seedlings expressing eGFP, Mlp124357-eGFP, Mlp124357GA-eGFP, AtPDI-11-eGFP or PtPDI-eGFP from 14-days-old plantlets, Figure S2: Mlp124357GA loses tonoplast localization in stable *Arabidopsis* transgenic line. Live-cell imaging of leaf epidermal cells of seven-day-old stable *Arabidopsis* eGFP, Mlp124357-eGFP or Mlp124357^GA^-eGFP transgenic lines, Figure S3: Distribution of Mlp124357-eGFP proteins in subcellular fractions. Cellular membrane fractions were obtained from fresh leaves of 3-week-old Col-0-eGFP and Mlp124357-eGFP plants by differential centrifugation and Mlp124357 was detected by Western hybridization with an antibody recognizing eGFP, Figure S4: Localization of Mlp124357 in tonoplast, bulbs, and TVSs does not change in the absence of AT2G33210, AT5G15650, and AT1G04040 genes in *Arabidopsis*. Live-cell imaging of leaf epidermal cells of the crossed lines between Mlp124357-eGFP with the indicated genes knock-out lines; SALK-120568, SALK-132152, or SALK-068225, Figure S5: Multiple sequence alignment of PDIs. The amino acid sequence of AtPDI-11, PtPDI, and MlpPDI was compared. Identical/highly conserved residues (*); semi-conserved residues (:) and conserved residues (.) are marked. The AtPDI-11 shows 87% and 30% of sequence identity with PtPDI and MlpPDI, respectively, Figure S6: Sub-cellular localization of AtPDI-11 or PtPDI in *N. benthamiana* epidermal cells. Laser-scanning confocal microscopy shows that AtPDI-11-eGFP (A,B) and PtPDI-eGFP (C,D) accumulate both in the tonoplast and endoplasmic reticulum (ER), Figure S7: Heatmap of differentially expressed genes in Col-0-eGFP and Mlp124357-eGFP based on transcriptome analysis. Table S1: List of putative interactor proteins of Mlp124357 from IP/MS, Table S2: List of up- and down-regulated genes from transcriptome analysis.

## References

[1] Dodds P.N., Rathjen J.P. (2010). Plant immunity: Towards an integrated view of plant–pathogen interactions. Nat. Rev. Genet..

[2] Ahmed M.B., dos Santos K.C.G., Sanchez I.B., Petre B., Lorrain C., Plourde M.B., Duplessis S., Desgagné-Penix I., Germain H. (2018). A rust fungal effector binds plant DNA and modulates transcription. Sci. Rep..

[3] Caillaud M.C., Piquerez S.J., Fabro G., Steinbrenner J., Ishaque N., Beynon J., Jones J.D. (2012). Subcellular localization of the Hpa RxLR effector repertoire identifies a tonoplast-associated protein HaRxL17 that confers enhanced plant susceptibility. Plant J..

[4] Petre B., Lorrain C., Saunders D.G., Win J., Sklenar J., Duplessis S., Kamoun S. (2016). Rust fungal effectors mimic host transit peptides to translocate into chloroplasts. Cell. Microbiol..

[5] Petre B., Saunders D.G., Sklenar J., Lorrain C., Win J., Duplessis S., Kamoun S. (2015). Candidate effector proteins of the rust pathogen Melampsora larici-populina target diverse plant cell compartments. Mol. Plant-Microbe Interact..

[6] Vargas W.A., Sanz-Martín J.M., Rech G.E., Armijos-Jaramillo V.D., Rivera L.P., Echeverria M.M., Díaz-Mínguez J.M., Thon M.R., Sukno S.A. (2016). A fungal effector with host nuclear localization and DNA-binding properties is required for maize anthracnose development. Mol. Plant-Microbe Interact..

[7] Schornack S., van Damme M., Bozkurt T.O., Cano L.M., Smoker M., Thines M., Gaulin E., Kamoun S., Huitema E. (2010). Ancient class of translocated oomycete effectors targets the host nucleus. Proc. Natl. Acad. Sci. USA.

[8] Wirthmueller L., Roth C., Fabro G., Caillaud M.C., Rallapalli G., Asai S., Sklenar J., Jones A.M., Wiermer M., Jones J.D. (2015). Probing formation of cargo/importin-α transport complexes in plant cells using a pathogen effector. Plant J..

[9] Dangl J.L., Horvath D.M., Staskawicz B.J. (2013). Pivoting the plant immune system from dissection to deployment. Science.

[10] Bombarely A., Rosli H.G., Vrebalov J., Moffett P., Mueller L.A., Martin G.B. (2012). A draft genome sequence of Nicotiana benthamiana to enhance molecular plant-microbe biology research. Mol. Plant-Microbe Interact..

[11] Germain H., Joly D.L., Mireault C., Plourde M.B., Letanneur C., Stewart D., Morency M.J., Petre B., Duplessis S., Séguin A. (2018). Infection assays in Arabidopsis reveal candidate effectors from the poplar rust fungus that promote susceptibility to bacteria and oomycete pathogens. Mol. Plant Pathol..

[12] Goodin M.M., Zaitlin D., Naidu R.A., Lommel S.A. (2008). Nicotiana benthamiana: Its history and future as a model for plant–pathogen interactions. Mol. Plant-Microbe Interact..

[13] Win J., Chaparro-Garcia A., Belhaj K., Saunders D., Yoshida K., Dong S., Schornack S., Zipfel C., Robatzek S., Hogenhout S. (2012). Effector biology of plant-associated organisms: Concepts and perspectives. Proc. Cold Spring Harb. Symp. Quant. Biol..

[14] Macho A.P., Guevara C.M., Tornero P., Ruiz-Albert J., Beuzon C.R. (2010). The Pseudomonas syringae effector protein HopZ1a suppresses effector-triggered immunity. New Phytol..

[15] Cui H., Wang Y., Xue L., Chu J., Yan C., Fu J., Chen M., Innes R.W., Zhou J.-M. (2010). Pseudomonas syringae effector protein AvrB perturbs Arabidopsis hormone signaling by activating MAP kinase 4. Cell Host Microbe.

[16] Anderson J.C., Pascuzzi P.E., Xiao F., Sessa G., Martin G.B. (2006). Host-mediated phosphorylation of type III effector AvrPto promotes Pseudomonas virulence and avirulence in tomato. Plant Cell.

[17] Nimchuk Z., Marois E., Kjemtrup S., Leister R.T., Katagiri F., Dangl J.L. (2000). Eukaryotic fatty acylation drives plasma membrane targeting and enhances function of several type III effector proteins from Pseudomonas syringae. Cell.

[18] Bai X., Correa V.R., Toruño T.Y., Ammar E.-D., Kamoun S., Hogenhout S.A. (2009). AY-WB phytoplasma secretes a protein that targets plant cell nuclei. Mol. Plant-Microbe Interact..

[19] Szurek B., Marois E., Bonas U., Van den Ackerveken G. (2001). Eukaryotic features of the Xanthomonas type III effector AvrBs3: Protein domains involved in transcriptional activation and the interaction with nuclear import receptors from pepper. Plant J..

[20] Duplessis S., Joly D.L., Dodds P.N. (2012). Rust effectors. Effectors in Plant-Microbe Interactions.

[21] Pennisi E. (2010). Armed and dangerous. Science.

[22] Duplessis S., Major I., Martin F., Séguin A. (2009). Poplar and pathogen interactions: Insights from Populus genome-wide analyses of resistance and defense gene families and gene expression profiling. Crit. Rev.Plant Sci..

[23] Hacquard S., Petre B., Frey P., Hecker A., Rouhier N., Duplessis S. (2011). The poplar-poplar rust interaction: Insights from genomics and transcriptomics. J. Pathog..

[24] Major I.T., Nicole M.-C., Duplessis S., Séguin A. (2010). Photosynthetic and respiratory changes in leaves of poplar elicited by rust infection. Photosynth. Res..

[25] Duplessis S., Cuomo C.A., Lin Y.-C., Aerts A., Tisserant E., Veneault-Fourrey C., Joly D.L., Hacquard S., Amselem J., Cantarel B.L. (2011). Obligate biotrophy features unraveled by the genomic analysis of rust fungi. Proc. Natl. Acad. Sci. USA.

[26] Hacquard S., Delaruelle C., Legué V., Tisserant E., Kohler A., Frey P., Martin F., Duplessis S. (2010). Laser capture microdissection of uredinia formed by Melampsora larici-populina revealed a transcriptional switch between biotrophy and sporulation. Mol. Plant-Microbe Interact..

[27] Hacquard S., Joly D.L., Lin Y.-C., Tisserant E., Feau N., Delaruelle C., Legué V., Kohler A., Tanguay P., Petre B. (2012). A comprehensive analysis of genes encoding small secreted proteins identifies candidate effectors in Melampsora larici-populina (poplar leaf rust). Mol. Plant-Microbe Interact..

[28] Saunders D.G., Win J., Cano L.M., Szabo L.J., Kamoun S., Raffaele S. (2012). Using hierarchical clustering of secreted protein families to classify and rank candidate effectors of rust fungi. PLoS ONE.

[29] Madina M.H., Zheng H., Germain H. (2018). New insight into bulb dynamics in the vacuolar lumen of Arabidopsis cells. Botany.

[30] Karimi M., Inzé D., Depicker A. (2002). GATEWAY™ vectors for Agrobacterium-mediated plant transformation. Trends Plant Sci..

[31] Sparkes I.A., Runions J., Kearns A., Hawes C. (2006). Rapid, transient expression of fluorescent fusion proteins in tobacco plants and generation of stably transformed plants. Nat. Protoc..

[32] Mireault C., Paris L.-E., Germain H. (2014). Enhancement of the Arabidopsis floral dip method with XIAMETER OFX-0309 as alternative to Silwet L-77 surfactant. Botany.

[33] Dong O.X., Meteignier L.V., Plourde M.B., Ahmed B., Wang M., Jensen C., Jin H., Moffett P., Germain H. (2016). *Arabidopsis* TAF15b Localizes to RNA Processing Bodies and Contributes to snc1-Mediated Autoimmunity. Mol. Plant Microbe Interact..

[34] Hammer O., Harper D.A.T., Ryan P.D. (2001). PAST: Paleontological statistics software package for education and data analysis. Paleontol. Electron..

[35] Germain H., Gray-Mitsumune M., Houde J., Benhamman R., Sawasaki T., Endo Y., Matton D.P. (2013). The Solanum chacoense ovary receptor kinase 11 (ScORK11) undergoes tissue-dependent transcriptional, translational and post-translational regulation. Plant Physiol. Biochem..

[36] Widell S., Larsson C. (1981). Separation of presumptive plasma membranes from mitochondria by partition in an aqueous polymer two-phase system. Physiol. Plant..

[37] Serino G., Deng X.W. (2007). Protein coimmunoprecipitation in *Arabidopsis*. CSH Prot..

[38] Nesvizhskii A.I., Keller A., Kolker E., Aebersold R. (2003). A statistical model for identifying proteins by tandem mass spectrometry. Anal. Chem..

[39] Germain H., Gray-Mitsumune M., Lafleur E., Matton D.P. (2008). ScORK17, a transmembrane receptor-like kinase predominantly expressed in ovules is involved in seed development. Planta.

[40] Bindea G., Galon J., Mlecnik B. (2013). CluePedia Cytoscape plugin: Pathway insights using integrated experimental and in silico data. Bioinformatics.

[41] Xu D., Zhang Y. (2012). Ab initio protein structure assembly using continuous structure fragments and optimized knowledge-based force field. Proteins Struct. Funct. Bioinform..

[42] Yang J., Yan R., Roy A., Xu D., Poisson J., Zhang Y. (2015). The I-TASSER Suite: Protein structure and function prediction. Nat. Methods.

[43] Kozakov D., Hall D.R., Xia B., Porter K.A., Padhorny D., Yueh C., Beglov D., Vajda S. (2017). The ClusPro web server for protein–protein docking. Nat. Protoc..

[44] Pierce B.G., Hourai Y., Weng Z. (2011). Accelerating protein docking in ZDOCK using an advanced 3D convolution library. PLoS ONE.

[45] Schneidman-Duhovny D., Inbar Y., Nussinov R., Wolfson H.J. (2005). PatchDock and SymmDock: Servers for rigid and symmetric docking. Nucleic Acids Res..

[46] Tovchigrechko A., Vakser I.A. (2006). GRAMM-X public web server for protein–protein docking. Nucleic Acids Res..

[47] DeLano W.L. (2002). The PyMOL Molecular Graphics System. http://www.pymol.org.

[48] Yuan S., Chan H.S., Hu Z. (2017). Using PyMOL as a platform for computational drug design. Wiley Interdiscip. Rev. Comput. Mol. Sci..

[49] Bronnimann M.P., Chapman J.A., Park C.K., Campos S.K. (2013). A transmembrane domain and GxxxG motifs within L2 are essential for papillomavirus infection. J. Virol..

[50] Cabanillas D.G., Jiang J., Movahed N., Germain H., Yamaji Y., Zheng H., Laliberte J.-F. (2018). Turnip mosaic virus uses the SNARE protein VTI11 in an unconventional route for replication vesicle trafficking. Plant Cell.

[51] Teese M.G., Langosch D. (2015). Role of GxxxG motifs in transmembrane domain interactions. Biochemistry.

[52] Yang J., Zhang Y. (2015). Protein Structure and Function Prediction Using I-TASSER. Curr. Protoc. Bioinform..

[53] Jones J.D., Dangl J.L. (2006). The plant immune system. Nature.

[54] Speth E.B., Lee Y.N., He S.Y. (2007). Pathogen virulence factors as molecular probes of basic plant cellular functions. Curr. Opin. Plant Biol..

[55] Cunnac S., Lindeberg M., Collmer A. (2009). Pseudomonas syringae type III secretion system effectors: Repertoires in search of functions. Curr. Opin. Microbiol..

[56] Torto T.A., Li S., Styer A., Huitema E., Testa A., Gow N.A., Van West P., Kamoun S. (2003). EST mining and functional expression assays identify extracellular effector proteins from the plant pathogen Phytophthora. Genome Res..

[57] Faris J.D., Zhang Z., Lu H., Lu S., Reddy L., Cloutier S., Fellers J.P., Meinhardt S.W., Rasmussen J.B., Xu S.S. (2010). A unique wheat disease resistance-like gene governs effector-triggered susceptibility to necrotrophic pathogens. Proc. Natl. Acad. Sci. USA.

[58] Liu Z., Zhang Z., Faris J.D., Oliver R.P., Syme R., McDonald M.C., McDonald B.A., Solomon P.S., Lu S., Shelver W.L. (2012). The cysteine rich necrotrophic effector SnTox1 produced by Stagonospora nodorum triggers susceptibility of wheat lines harboring Snn1. PLoS Pathog..

[59] Brar H.K., Swaminathan S., Bhattacharyya M.K. (2011). The Fusarium virguliforme toxin FvTox1 causes foliar sudden death syndrome-like symptoms in soybean. Mol. Plant-Microbe Interact..

[60] Shang Y., Li X., Cui H., He P., Thilmony R., Chintamanani S., Zwiesler-Vollick J., Gopalan S., Tang X., Zhou J.-M. (2006). RAR1, a central player in plant immunity, is targeted by Pseudomonas syringae effector AvrB. Proc. Natl. Acad. Sci. USA.

[61] Kanneganti T.-D., Huitema E., Cakir C., Kamoun S. (2006). Synergistic interactions of the plant cell death pathways induced by Phytophthora infestans Nep1-like protein PiNPP1.1 and INF1 elicitin. Mol. Plant-Microbe Interact..

[62] Chang H.-X., Domier L.L., Radwan O., Yendrek C.R., Hudson M.E., Hartman G.L. (2016). Identification of multiple phytotoxins produced by Fusarium virguliforme including a phytotoxic effector (FvNIS1) associated with sudden death syndrome foliar symptoms. Mol. Plant-Microbe Interact..

[63] Madina M.H., Rahman M.S., Zheng H., Germain H. (2019). Vacuolar membrane structures and their roles in plant-pathogen interactions. Plant Mol. Biol..

[64] Verchot J. (2012). Cellular chaperones and folding enzymes are vital contributors to membrane bound replication and movement complexes during plant RNA virus infection. Front. Plant Sci..

[65] Yuen C., Matsumoto K., Christopher D. (2013). Variation in the subcellular localization and protein folding activity among Arabidopsis thaliana homologs of protein disulfide isomerase. Biomolecules.

[66] Cho E.J., Yuen C.Y., Kang B.-H., Ondzighi C.A., Staehelin L.A., Christopher D.A. (2011). Protein disulfide isomerase-2 of Arabidopsis mediates protein folding and localizes to both the secretory pathway and nucleus, where it interacts with maternal effect embryo arrest factor. Mol. Cells.

[67] Ondzighi C.A., Christopher D.A., Cho E.J., Chang S.-C., Staehelin L.A. (2008). Arabidopsis protein disulfide isomerase-5 inhibits cysteine proteases during trafficking to vacuoles before programmed cell death of the endothelium in developing seeds. Plant Cell.

[68] Wittenberg G., Levitan A., Klein T., Dangoor I., Keren N., Danon A. (2014). Knockdown of the A rabidopsis thaliana chloroplast protein disulfide isomerase 6 results in reduced levels of photoinhibition and increased D 1 synthesis in high light. Plant J..

[69] Turano C., Coppari S., Altieri F., Ferraro A. (2002). Proteins of the PDI family: Unpredicted non-ER locations and functions. J. Cell Physiol..

[70] Rovenich H., Boshoven J.C., Thomma B.P. (2014). Filamentous pathogen effector functions: Of pathogens, hosts and microbiomes. Curr. Opin. Plant Biol..

[71] Coaker G., Falick A., Staskawicz B. (2005). Activation of a phytopathogenic bacterial effector protein by a eukaryotic cyclophilin. Science.

[72] Coaker G., Zhu G., Ding Z., Van Doren S.R., Staskawicz B. (2006). Eukaryotic cyclophilin as a molecular switch for effector activation. Mol. Microbiol..

[73] Kong G., Zhao Y., Jing M., Huang J., Yang J., Xia Y., Kong L., Ye W., Xiong Q., Qiao Y. (2015). The activation of Phytophthora effector Avr3b by plant cyclophilin is required for the nudix hydrolase activity of Avr3b. PLoS Pathog..

